# IL-1β- and IL-4-polarized macrophages have opposite effects on adipogenesis of intramuscular fibro-adipogenic progenitors in humans

**DOI:** 10.1038/s41598-018-35429-w

**Published:** 2018-11-19

**Authors:** C. Moratal, J. Raffort, N. Arrighi, S. Rekima, S. Schaub, C. A. Dechesne, G. Chinetti, C. Dani

**Affiliations:** 1 Université Côte d’Azur, CNRS, Inserm, iBV, Nice, France; 2 Université Cote d’Azur, Inserm, C3M, Nice, France; 3 Clinical Chemistry Laboratory, University Hospital of Nice, Nice, France

## Abstract

Intramuscular fat deposition represents a negative prognostic factor for several myopathies, metabolic diseases and aging. Fibro-adipogenic progenitors (FAPs) are considered as the main source of intramuscular adipocytes, but the mechanisms controlling their adipogenic potential are still not elucidated in humans. The aim of this study was to explore the regulation of human FAP adipogenesis by macrophages. We found that CD140a-expressing FAPs were located close to CD68 positive macrophages in muscles from patients with Duchenne muscular dystrophy (DMD). This strongly suggests a potential interaction between FAPs and macrophages *in vivo*. Isolated human primary FAPs were then differentiated in the presence of conditioned media obtained from primary blood monocyte-polarized macrophages. Molecules released by IL-1β-polarized macrophages (M(IL-1β)) drastically reduced FAP adipogenic potential as assessed by decreased cellular lipid accumulation and reduced gene expression of adipogenic markers. This was associated with an increased gene expression of pro-inflammatory cytokines in FAPs. Conversely, factors secreted by IL-4-polarized macrophages (M(IL-4)) enhanced FAP adipogenesis. Finally, the inhibition of FAP adipocyte differentiation by M(IL-1β) macrophages requires the stimulation of Smad2 phosphorylation of FAPs. Our findings identify a novel potential crosstalk between FAPs and M(IL-1β) and M(IL-4) macrophages in the development of adipocyte accumulation in human skeletal muscles.

## Introduction

Under pathological conditions such as Duchenne muscular dystrophy (DMD)^1,2^, type II diabetes^3,4^, sarcopenia^5^, denervation^6,7^, and chronic disease-induced muscle atrophy^8,9^, intramuscular adipocytes invade skeletal muscles and replace a large proportion of muscle fibers. This adipocyte infiltration leads to a poor quality of muscles and dysfunctional performances^10^. Fatty degeneration was shown to be an accurate measurement of the severity of DMD and a limit for the success of current cell and genetic therapies. Many cells can contribute to ectopic intramuscular adipocyte deposits including myoendothelial cells^11,12^, pericytes^13^, mesoangioblasts^14,15^ and PW1-expressing cells (PICs)^16,17^. Recent studies demonstrated that intramuscular adipocytes mainly emanated from a population of fibro-adipogenic progenitors (FAPs) that reside between muscle fibers^18,19^. Their role in muscle regeneration was in part elucidated in mice. After injury, FAPs proliferate, interact with myoblasts to promote the formation of new muscle fibers^18^, and eventually return to quiescent state or are cleared by apoptosis^20^. With impaired regeneration as in DMD, FAPs expand and differentiate to generate the components of the fibro-fatty tissue^21,22^. We and others have identified the presence of FAPs in human skeletal muscles by the expression of the specific markers CD140a, CD15 and CD34^23–26^. *In vitro* and *in vivo*, these progenitors differentiate into adipocytes and myofibroblasts in response to adequate stimuli^27,28^. While Uezumi *et al*. reported an inhibition of FAP adipogenesis by myoblasts in mice^19^, the regulation of FAP differentiation during muscle regeneration remains to be elucidated in humans.

Among potential regulators of intramuscular adiposity are macrophages whose depletion alters muscle regeneration^29,30^. In particular, the ablation of CD11b-positive macrophages in mice affects the fat accumulation in injured muscles^31^. Conditioned medium from murine macrophages inhibits the adipogenic potential of a subpopulation of multipotent muscle stem cells^32^. Moreover, molsidomine, a nitric oxide donating drug, modulator of the macrophage recruitment in damaged muscles, reduces the deposition of both skeletal muscle fat and connective tissues in a mouse model of DMD^22^. Interestingly, an interaction between macrophages and FAPs during mouse muscle regeneration has recently been shown^20^. Indeed, infiltrating inflammatory macrophages directly induce apoptosis of FAPs *via* tumor necrosis factor α (TNFα) secretion. However, in chronic damage, macrophages expressing high level of tumor growth factor β1 (TGFβ1) prevent the apoptosis of FAPs and induce their fibrogenic potential. Our working hypothesis is that macrophage infiltration affects fat expansion through a paracrine action on adipocyte differentiation of human FAPs.

Macrophages can adopt various functional phenotypes and activation states depending on their environment^33^. Very schematically, at least two populations of macrophages have been described according to their inflammatory status. While pro-inflammatory or classically activated M1 macrophages are induced by TH1 cytokines like interferon γ (IFNγ) or interleukin 1β (IL-1β), alternatively activated M2 macrophages are triggered by TH2 cytokines such as IL-4 and IL-13. However, such activation profile has never been demonstrated in human skeletal muscles^34^. The sequential infiltration of pro-inflammatory and anti-inflammatory macrophages in mouse injured muscles shows their differential roles during muscle regeneration. Pro-inflammatory macrophages invade the injured areas shortly after damage to release a number of cytokines that stimulate myoblast proliferation. Anti-inflammatory macrophages are present locally at late stages of regeneration and act as promoters of myoblast differentiation and fusion^35^.

While the regulation of myogenic lineage by macrophages is well characterized^36^, the control of FAP differentiation by macrophages is still not investigated in humans. Moreover, the recent study of interactions between mouse FAPs and macrophages has not clearly distinguished the impacts of different macrophage functional phenotypes on FAP fibro-adipogenesis^20^. Here we compare the influence of factors secreted by human healthy donor primary IL-1β-polarized macrophages (M (IL-1β)) or IL-4-induced macrophages (M(IL-4)) on the differentiation of FAPs derived from human skeletal muscle. This crosstalk between FAPs and macrophages uncovers a new mechanism regulating the intramuscular adipocyte deposits in human skeletal muscles.

## Results

### FAPs and macrophages closely located in human dystrophic muscles

**Figure 1:**
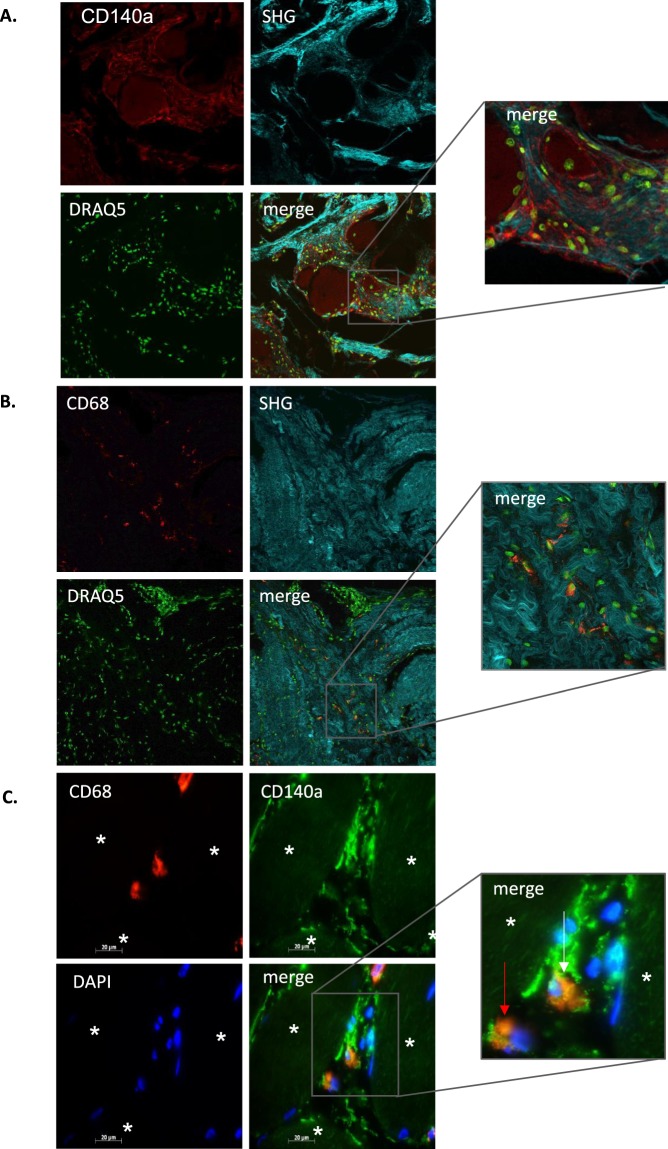
FAPs closely localized with CD68^+^ macrophages within degenerating areas of DMD muscles. Frozen sections of DMD biopsies were stained with anti-CD140a for detection of FAPs *(A)* or anti-CD68 antibody for macrophages (**B**) or both (**C**). Fibrillar collagen was visualized in blue by second-harmonic generation imaging (SHG) and DNA was stained with DRAQ5 in (**A**,**B**) (**C**) Cell nuclei were visualized with DAPI (blue). White arrow shows one CD68^+^ cell in contact with one FAP, and red arrow shows another CD68^+^ cell in proximity to one FAP. Myofibers are indicated by white asterisks. Scale bar: 20 µm. The right panels are a magnification of the merge panels. The analysis was performed on three different DMD biopsies. Representative views are shown.

DMD muscles are characterized by a significant inflammatory reaction with an increased number of infiltrating macrophages^37^ and FAPs^27^. Indeed, DMD muscles presented numerous CD140^+^ FAPs (Fig. 1A) and macrophages expressing CD68 (Fig. 1B), a surface marker expressed by all macrophage subtypes^38^. By contrast, healthy muscles displayed few CD140^+^ FAPs (Supplementary Fig. S1A) and CD68^+^ macrophages (Supplementary Fig. S1B). Interestingly, CD140^+^ FAPs (Fig. 1A) and CD68^+^ macrophages (Fig. 1B) were localized in the same regions where degenerating areas were observed, as represented by the presence of fibrillary collagen stained by SHG (second harmonic generation) in blue. To demonstrate a possible connection between FAPs and macrophages, DMD muscle sections were co-stained with anti-CD140a and anti-CD68 antibodies. Importantly, CD140^+^ FAP and CD68^+^ macrophages were found in very close proximity in degenerating areas (Fig. 1C). At the higher magnification, we observed that one CD68^+^ macrophage (white arrow) was in contact with FAPs (in green), whereas another CD68^+^ macrophages (red arrow) was located less than 20 µm from FAPs (Fig. 1C, right panel). The number of CD68^+^ macrophages *per* section was approximately 12.4 ± 6.85 and the number of CD68^+^ macrophages in contact with FAPs *per* section was approximately 3 to 8 ± 0.10. This results show for the first time in humans and particularly in DMD muscles, that FAPs and macrophages resided near to each other, suggesting a potential *in vivo* interaction between these two types of cells.

### Unlike fibrogenesis, adipocyte differentiation of human FAPs is affected by M(IL-1β) and M(IL-4) macrophage-secreted factors

Before evaluating the effect of macrophage-derived factors on human FAP differentiation, we firstly validated the *in vitro* model of human primary macrophage polarization as well as the adipogenic potential of FAPs. Three distinct conditioned media (CM) from control unpolarized resting macrophages (RM), IL-1β-treated (M(IL-1β)) or IL-4-treated (M(IL-4)) macrophages were produced. IL-1β, but not IL-4, increased the gene expression of *IL1B*, *IL6* and *CCL2* in macrophages (Supplementary Fig. S2A). IL-4, but not IL-1β, stimulated the gene expression of *MRC1*, *CD200R1*, *F13A1* and *CCL18* (Supplementary Fig. S2B) in macrophages. According to the nomenclature of macrophage activation and polarization^33^, these data indicate that M(IL-1β) and M(IL-4) can be considered as pro-inflammatory and anti-inflammatory macrophages, respectively. RM CM was used as control CM for unpolarized RM macrophages. We also confirmed the high adipogenic potential of FAPs as shown by the presence of adipocytes with lipid droplets (Supplementary Fig. S3A) and the induction of adipocyte marker expression (Supplementary Fig. S3B) after 3, 8, 13, 17 and 20 days of differentiation in the presence of a pro-adipogenic medium.

**Figure 2:**
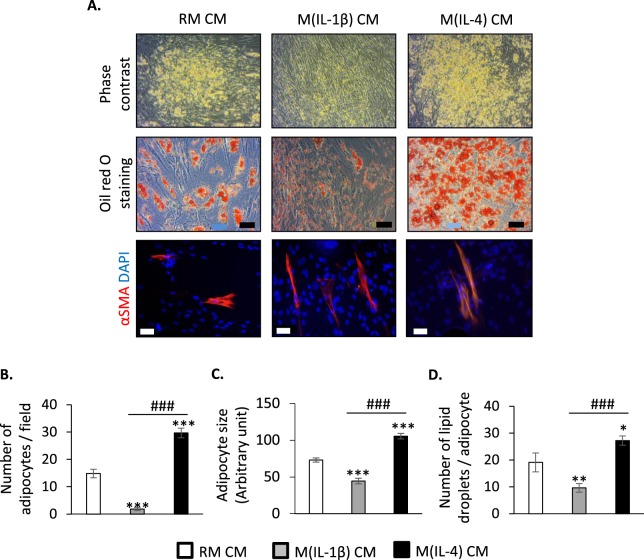
Factors secreted by M(IL-1β) and M(IL-4) macrophages differently affect human FAP adipogenesis. FAPs were differentiated into adipocytes for 10 days in a pro-adipogenic medium containing conditioned medium (CM) from RM, M(IL-1β) or M(IL-4) macrophages. (**A**) Adipocytes were unstained (top panel) or stained by oil Red O (middle panel). Pictures were captured under light microscopy (scale bar: 10 µm). In the bottom panel, FAP-derived myofibroblasts were visualized by αSMA (α smooth muscle actin) immuno-staining (red). Nuclei were labelled with DAPI in blue (scale bar: 50 µm). The number of adipocytes *per* field (**B**), the adipocyte size (**C**), and the number of lipid droplets *per* adipocyte (**D**) were measured on oil red O-stained cells. One representative experiment of nine separate experiments was shown (3 blood donors and 3 muscle donors). Data are presented as means of triplicates ± SEM; * indicates statistically significant difference *vs* RM CM-treated FAPs; # indicates statistically significant difference *vs* M(IL-1β) CM-treated FAPs; *P < 0.05, **P < 0.01, *** or ^###^*P* < 0.001.

To note, adipogenesis is conserved in FAPs isolated from skeletal muscles of DMD patients. Indeed, gene expression of adipogenic markers *PLIN1*, *ADIPOQ* and *FABP4* was similar in DMD patients compared to healthy donors (Supplementary Fig. S3C). Then, confluent FAPs were induced to differentiate in a pro-adipogenic medium containing RM, M(IL-1β) or M(IL-4) CM (50/50), and adipogenic differentiation was measured 10 days later. Interestingly, CM from M(IL-1β) or M(IL-4) macrophages strongly affected FAP adipocyte differentiation compared to RM CM (Fig. 2A, top and middle panels). Indeed, lipid accumulation, as stained by oil red O, was lower in the presence of CM from M(IL-1β) macrophages, compared to RM CM. By contrast, lipid accumulation increased after incubation with CM from M(IL-4) macrophages. In line, the number of FAPs committed into adipogenesis decreased in the presence of M(IL-1β) CM but strongly increased with CM from M(IL-4) macrophages (Fig. 2B). Moreover, a smaller size was observed for adipocytes differentiated with M(IL-1β) CM whereas bigger adipocytes were detected in the presence of M(IL-4) CM, compared to RM CM (Fig. 2C). This observation correlated with the lower number of lipid droplets/adipocyte observed with M(IL-1β) CM than with CM from M(IL-4) macrophages (Fig. 2D).

As we recently published^28^, FAPs isolated from healthy skeletal muscles have a fibrogenic potential. Thus, the effect of conditioned media on FAP fibrogenesis was assessed. After 10 days of differentiation in a pro-adipogenic medium, only few alpha smooth muscle actin (αSMA)-positive myofibroblasts were observed in the presence of RM, M(IL-1β) and M(IL-4) CM (Fig. 2A, bottom panel), suggesting that the inflammatory status of macrophages did not control FAP differentiation into myofibroblasts.

### M(IL-1β) and M(IL-4) macrophage-secreted factors alter gene expression in human FAPs

**Figure 3:**
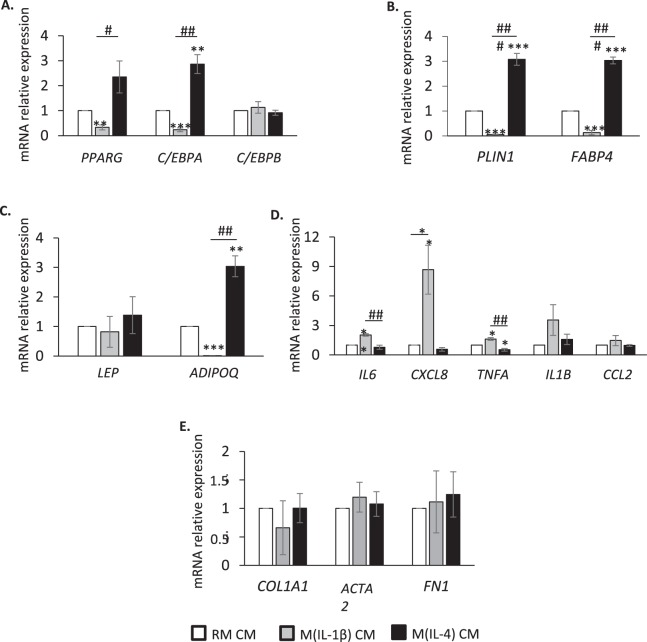
Conditioned media from M(IL-1β) and M(IL-4) macrophages alter gene expression in human FAPs. FAPs were differentiated into adipocytes for 10 days in a pro-adipogenic medium containing RM (white bars), M(IL-1β) (grey bars) or M(IL-4) (dark bars) CM. Expression of adipose transcriptional factors (**A**), their target genes (**B**), adipokines (**C**), pro-inflammatory (**D**), and fibrogenic markers (**E**) was measured by quantitative Q-PCR. Data are presented as means ± SEM of three separate experiments performed in duplicate (1 blood donor and 3 muscle donors); * indicates statistically significant difference *vs* RM CM-treated FAPs; # indicates statistically significant difference *vs* M(IL-1β) CM-treated FAPs; * or ^#^*P* < 0.05, ** or ^##^*P* < 0.01, *** or ^###^*P* < 0.001. PPARG: peroxisome proliferator-activated receptor γ; C/EBPA and C/EBPB: CCAAT/enhancer-binding protein α and β; PLIN1: perilipin 1; FABP4: fatty acid binding protein 4; LEP: leptin; ADIPOQ: adiponectin; IL1B and IL6: interleukin-1β and -6; CXCL8: C-X-C motif chemokine ligand 8; TNFA: tumor necrosis factor α; CCL2: C-C motif chemokine ligand 2; COL1A1: collagen 1 type A1; ACTA2: actin α 2; FN1: fibronectin 1.

To elucidate the molecular mechanisms involved in the differential regulation of FAP adipogenesis by M(IL-1β) and M(IL-4) CM, gene expression of several specific adipose markers was analysed. Expression of the transcription factors peroxisome proliferator-activated receptor γ (*PPARG*) and CCAAT/enhancer-binding protein α (*C/EBP)A* (Fig. 3A) and their target genes perilipin 1 (*PLIN1*) and fatty acid binding protein 4 (*FABP4*) (Fig. 3B) strongly diminished in M(IL-1β) CM-treated FAPs while significantly increased in M(IL-4) CM-treated FAPs compared to RM CM-treated FAPs. However, the expression of *C/EBPB* was unchanged in the presence of M(IL-1β) or M(IL-4) CM (Fig. 3A). The expression of adiponectin (*ADIPOQ*) also decreased with M(IL-1β) CM while it increased upon M(IL-4) CM treatment (Fig. 3C). Of note, M(IL-1β) and M(IL-4) CM did not affect leptin (*LEP*) expression (Fig. 3C).

The effect of M(IL-1β) and M(IL-4) CM on the inflammatory status of FAPs after 10 days of differentiation was also investigated by measuring the expression of pro-inflammatory markers (Fig. 3D). Interestingly, only M(IL-1β) CM significantly induced *IL*6, C-X-C motif chemokine ligand 8 (*CXCL8*) and *TNFA* expression compared to RM or M(IL-4) CM. *IL1B* and C-C motif chemokine ligand 2 (*CCL2*) expression also tended to be more expressed in M(IL-1β) CM-treated FAPs but without reaching statistical significance.

Finally, in line with the results of fibrogenesis (Fig. 2A bottom panel), expression of specific fibrogenic markers such as collagen 1 type A1 (*COL1A1)*, fibronectin 1 (*FN1*) and α actin 2 (*ACTA2)* did not vary between the three experimental conditions (Fig. 3E).

Therefore, M(IL-1β) macrophage-secreted factors inhibited adipogenesis and promoted the gene pro-inflammatory profile while M(IL-4) macrophage-secreted factors stimulated adipocyte differentiation of human FAPs.

### Smad2 phosphorylation drives the reduced FAP adipogenesis by M(IL-1β) macrophages

**Figure 4:**
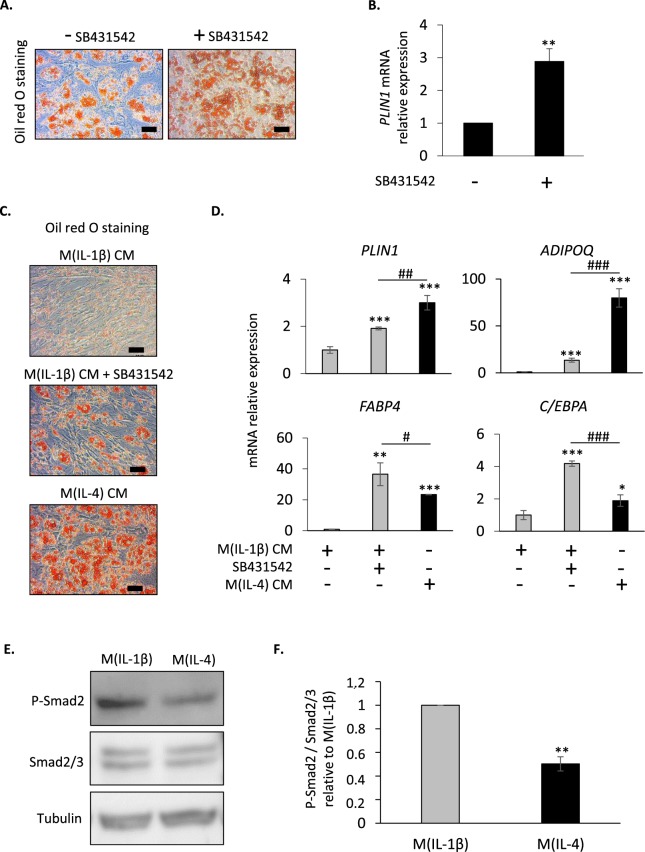
The M(IL-1β)-dependent inhibition of FAP adipogenesis requires Smad2 phosphorylation. (**A**) FAPs were differentiated into adipocytes for 10 days in a pro-adipogenic medium with or without SB431542. Oil red O-stained adipocytes were visualized by light microscopy. Scale bar: 10 µm. (**B**) *PLIN1* gene expression was measured by quantitative Q-PCR. Data are presented as means ± SEM of three separate experiments in duplicates; ** *P* < 0.01 *vs* untreated FAPs. (**C**,**D**) FAPs were differentiated into adipocytes for 10 days in a pro-adipogenic medium containing M(IL-1β) CM with or without SB431542 or containing M(IL-4) CM. (**C**) Oil red O-stained adipocytes were visualized by light microscopy. Scale bar: 10 µm. (**D**) *PLIN1*, *ADIPOQ*, *FABP4 and C/EBPA* gene expression was determined by quantitative Q-PCR. Data are presented as means ± SD of one representative experiment performed in triplicates. * indicates statistically significant difference *vs* M1 CM-treated FAPs; # indicates statistically significant difference *vs* FAPs treated with M(IL-1β) CM and SB431542; * or ^#^*P* < 0.05, ** or ^##^*P* < 0.01, *** or ^###^*P* < 0.001. (**E**,**F**) Confluent FAPs were treated with a pro-adipogenic medium containing M(IL-1β) CM or M(IL-4) CM and total proteins were extracted 1 hour later. (**E**) Protein expression of phosphorylated Smad2 (P-Smad2), total Smad2/3 and tubulin was assessed by western blot. (**F**) P-Smad2 band intensity was quantified and normalized to total Smad2/3 signals. ** *P* < 0.01 *vs* M(IL-1β) CM-treated FAPs.

Since an anti-adipogenic role of recombinant TGFβ1 has been previously shown in FAPs^28^, the effect of SB431542, a specific inhibitor of the TGFβ superfamily type I activin receptor-like kinase (ALK) receptors ALK4, ALK5, and ALK7^39^, was first examined on intrinsic FAP adipogenic differentiation. As expected, SB431542 stimulated FAP differentiation into adipocytes (Fig. 4A), an effect correlated with a three fold increase of *PLIN1* gene expression (Fig. 4B). These data prompted us to hypothesize a potential role of the ALK signalling pathway in the inhibition of adipogenic differentiation of FAPs by M(IL-1β) macrophages. To verify this hypothesis, FAPs were differentiated in the presence of M(IL-1β) CM with or without SB431542 and the effect of the inhibitor on adipogenesis was evaluated 10 days later and was compared to M(IL-4) CM-treated FAPs. The addition of SB431542 counteracted the M(IL-1β) CM-dependent inhibition of FAP adipogenesis, as assessed by an increase of oil red O staining (Fig. 4C) and the expression of several adipocyte markers (Fig. 4D). Lipid accumulation, *PLIN1* and *ADIPOQ* gene expression indicated that adipogenic rate in FAPs treated with M(IL-1β) CM and SB431542 was lower than in M(IL-4) CM-treated FAPs. By contrast, *FABP4* and *C/EBPA* expression was higher. We concluded that activation of the ALK/Smad2 pathway by soluble molecules secreted by M(IL-1β) macrophages was required to inhibit FAP differentiation into adipocytes.

We then measured the expression and phosphorylation of Smad2, a downstream effector of ALK. A lower Smad2 phosphorylation was observed in FAPs cultured with M(IL-4) CM, compared to M(IL-1β) CM-treated FAPs (Fig. 4E,F). This indicates that the regulation of FAP adipogenesis by M(IL-1β) and M(IL-4) macrophages was associated with a differential activation of the ALK/Smad2 signalling pathway.

### FAP localise closed to alternative macrophages in DMD muscles

**Figure 5:**
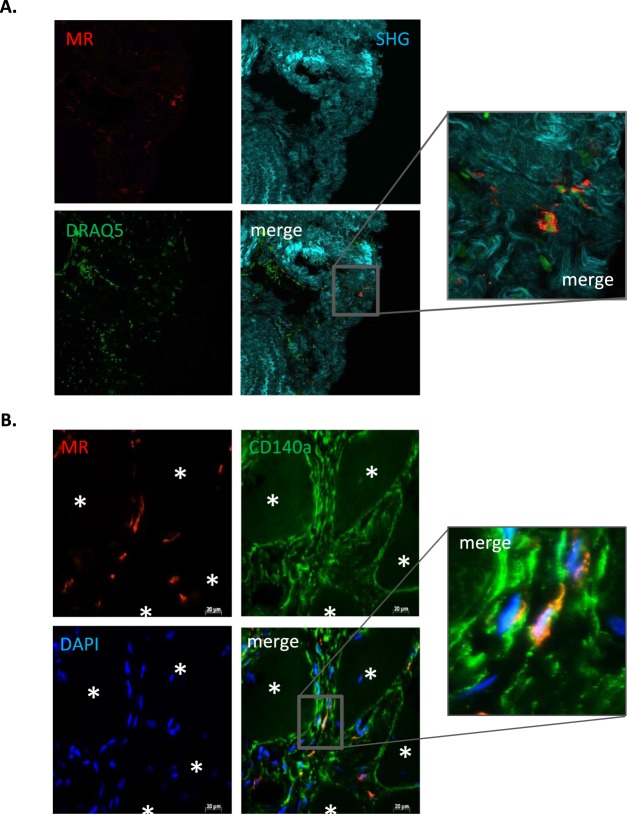
FAPs closely localized with MR^+^ macrophages in DMD muscles. (**A**) Frozen sections of DMD muscle biopsies were stained with anti-MR antibody to detect alternative macrophages (red). Fibrillar collagen was visualized in blue by second-harmonic generation imaging (SHG) and DNA was stained with DRAQ5 (green). (**B**) DMD muscle sections were co-stained with anti-MR antibody (red) and anti-CD140 antibody (green) to detect alternative macrophages and FAPs, respectively. Cell nuclei were visualized with DAPI (blue). Scale bar: 20 µm. The right panels are a magnification of the merge panels. The analysis was performed with three different DMD biopsies. Representative views are shown. MR: mannose receptor.

Expression of mannose receptor C-type 1 (*MRC1*), that codes for the mannose receptor (MR), was strongly induced in macrophages by IL-4 but not by IL-1β (Supplementary Fig. 2B). This receptor is considered as an *in vivo* marker for alternative macrophages^38^. We found the presence of MR^+^ macrophages in DMD muscle sections (Fig. 5A) and in healthy muscles (Supplementary Fig. S4). Interestingly, MR^+^ macrophages were located in the same fibrotic regions (Fig. 5A) than CD140^+^ FAPs (Fig. 1A) in DMD muscles. To assess a possible interaction between FAPs and MR^+^ macrophages *in vivo*, DMD muscle sections were co-stained with human anti-CD140a and anti-MR antibodies. Importantly, MR-positive macrophages were located near to the CD140^+^ FAPs (Fig. 5B). Indeed, at the higher magnification, we found one MR^+^ macrophage (in red) in contact with FAPs (in green) (Fig. 5B, right panel). The number of MR^+^ macrophages *per* section was approximately 4.25 ± 1.73 and the number of MR^+^ macrophages in contact with FAPs *per* section was approximately 1/2 ± 0.18. These data strongly suggest that *in vivo* M(IL-4) alternative macrophages could interact with FAPs to control their adipogenic potential.

## Discussion

We have earlier reported that FAPs located between myofibers in human skeletal muscles can differentiate into white adipocytes *in vitro* and *in vivo*^28^. The regulation of their adipogenesis during muscle regeneration was partly characterized in mice. Indeed, satellite cell-derived myofibers strongly inhibit adipogenesis of FAPs to prevent fatty infiltration in regenerating muscles toward successful repair^19^. In addition to myogenic progenitors and FAPs, macrophages are central actors of regeneration in healthy and dystrophic muscles^40^. However, to our knowledge, no studies on the control of FAP differentiation by macrophages have been performed in humans. Here, we report that IL-1β-activated macrophages and IL-4-polarized macrophages have opposite effects on FAP differentiation into adipocytes *in vitro*, and this was dependent on the Smad2 phosphorylation status in FAPs.

First, we show that CD140^+^ FAPs localized closed to CD68^+^ macrophages in DMD muscles, thus strengthening our hypothesis that macrophages could regulate FAPs behavior in humans. To verify this hypothesis, we used an experimental model where human FAPs were differentiated in the presence of conditioned medium from human blood monocyte-derived macrophages. Monocytes were differentiated for 6 days into unpolarized (RM) macrophages or were polarized into anti-inflammatory macrophages with IL-4 (M(IL-4)) or into pro-inflammatory macrophages with IL-1β (M(IL-1β)). The choice of these stimuli is consistent with the high IL1-β expression in the diaphragm of *mdx* mice^41^, an animal model to study DMD, and with the expression of IL-4 receptor by macrophages in quadriceps of these mice^42^. These results thus validate the pertinence of our *in vitro* model of macrophage polarization. We use CM from resting macrophages as control to assess the effect of macrophage inflammatory status on FAP differentiation and to get rid of the decrease in medium nutrients in M(IL-1β) and M(IL-4) CM.

Importantly, we demonstrated that factors released from M(IL-1β) macrophages inhibit the differentiation capacity of FAPs while M(IL-4) macrophage-secreted molecules have a pro-adipogenic effect. CM affect both the differentiation of FAPs into adipocytes, as indicated by the variation of adipocyte number, and the terminal adipogenesis of FAPs, as shown by the modulation of adipocyte size and the number of lipid droplets *per* adipocyte. We can hypothesize that the M(IL-4)-dependent increase of adipocyte number can be explained by an enhancement of FAP commitment towards the adipogenic lineage. Interestingly, *C/EBPB*, a master gene of adipocyte commitment^43^, was unaffected, suggesting that macrophages regulate events downstream to *C/EBPB* expression.

In the last decade, several studies reported that human macrophage cell lines or primary monocyte-derived pro-inflammatory macrophages inhibit adipogenesis of adipose progenitors derived from human adipose tissue^44–47^. Similar to these results, gene expression of *PPARG* and *C/EBPA*, the two master regulators of adipocyte differentiation^43^, decreased in FAPs cultured with M(IL-1β) CM. By contrast, since *C/EBPB* expression was unaffected, this suggests that this transcription factor is not required for the effect of macrophages on *PPARG* and *C/EBPA* gene expression at day 10 of differentiation. This was consistent with the reported anti-adipogenic effects of secreted factors from bacterial lipopolysaccharide (LPS)-activated human monocyte-derived macrophages on subcutaneous adipose tissue preadipocytes^47^. Conversely to Lacasa *et al*., leptin expression did not vary in our experiments in FAPs cultured with M(IL-1β) conditioned medium, suggesting a specificity of FAP-derived adipocytes. We had also shown that these adipocytes have the unexpected feature of being insulin-resistant^28^.

It was reported that M(IL-4) anti-inflammatory macrophages serve as an important source of catecholamine for beiging activation in mouse subcutaneous adipose tissue^48–51^. However, no expression of uncoupling protein 1 (*UCP1*), a marker of adipocyte beiging, was detected in human FAPs cultured with M(IL-4) conditioned medium (data not shown), suggesting that factors released by M(IL-4) macrophages stimulated the differentiation of FAPs into white adipocytes exclusively. This is in accordance with our previous work showing that human FAPs give rise to *bona fide* white adipocytes^28^.

We also found that anti-adipogenic effect of M(IL-1β) macrophages is associated with the increase of *IL-6*, *IL-8*, *TNFA*, *IL-1B* and *MCP-1* gene expression in FAPs after 10 days of culture in pro-adipogenic medium. Even if these results have to be confirmed at the protein level, the significance increase of pro-inflammatory cytokines in FAPs cultured with M(IL-1β) CM and their defective engagement toward adipogenesis needs to be understood, particularly during the muscle regeneration process and in DMD muscles characterized by macrophage infiltration in injured areas. Previous studies have reported that IL-6, MCP-1, TNFα and IL-1β are usually up-regulated in damaged muscle in the early phase of regeneration^52,53^ and in dystrophic muscles^54–56^ where an intramuscular accumulation of FAPs is observed. FAPs could be one of the sources of these cytokines in injured and diseased muscles and, thus, could mediate the inflammatory response. In addition to their role in inflammation, IL-6 and TNFα can also contribute to muscle homeostasis by controlling the proliferative and differentiation capacities of muscle stem cells^52,57^. Interestingly, mouse FAPs provide a source of IL-6 that regulate satellite cell activity^58^.

In addition to their adipogenic potential, *in vitro* human FAPs display fibrogenic capacity in response to TGFβ1^27,28^. We showed that M(IL-1β) and M(IL-4) macrophage-secreted factors did not affect extra-cellular matrix. In contrary to our results, it has been reported that inflammatory adipose tissue-derived preadipocytes exposed to macrophage-secreted factors overexpressed extra-cellular matrix genes^45,59,60^. This discrepancy could be explained by the different cytokines secreted by macrophages employed in each study, which differ in their origin (isolated from blood^45,59^ or adipose tissue^60^), and in their culture conditions (such as difference in stimuli used to induce differentiation). Moreover, our results were obtained in a pro-adipogenic medium, which is not representative of the environment of fibrotic muscles, such as DMD. Further experiments using for example a pro-fibrogenic medium complemented with TGFβ1, could allow concluding regarding a potential regulation of FAP fibrogenesis by macrophages.

Mechanistically, we show that M(IL-1β) macrophages-secreted factors highly phosphorylated Smad2 in FAPs, and this phosphorylation is mediated by ALK4/5/7 receptor activity. Human DMD muscles contain CD140a-expressing cells that are positive for phospho-Smad2/3^61^, but are also invaded by adipocytes. These conflicting results could be explained by the difference in the inflammatory status between *in vitro* and *in vivo* macrophages. Members of the TGFβ family, that are known to stimulate Smad2 phosphorylation *via* ALK4/5/7 receptor activity, are numerous such as activins, myostatine, GDF11 (growth differentiation factor 11) and TGFβs^62^. Their implication in the inhibition of FAP adipogenesis by M(IL-1β) macrophages remains to be identified. Finally, we detected FAPs in proximity to MR-positive macrophages in human DMD muscles suggesting that these macrophages could promote the accumulation of intramuscular adipocytes by interacting with FAPs. Furthermore, healthy and DMD FAPs have similar adipogenic differentiation rate suggesting that DMD FAPs could be competent to respond to signals issued from MR-positive macrophages.

In summary, our results show that M(IL-1β) macrophages release cytokines that inhibit FAP adipogenesis *via* Smad2 phosphorylation, whereas M(IL-4) macrophages have a pro-adipogenic effect. These differential interplays between FAPs and macrophage subtypes provide new elements to help developing medical regenerative strategies to prevent adipocyte deposits in diseased muscles associated with a chronic inflammation such as DMD.

## Materials and Methods

### Reagents and antibodies

Cell culture media, serum, phosphate-buffered saline and trypsin were purchased from Lonza (Verviers, Belgium) and cell culture reagents were from Sigma-Aldrich Chimie (Saint-Quentin Fallavier, France).

Human antibodies for immunofluorescence analysis were purchased as indicated: anti-αSMA (#A5228) from Sigma-Aldrich, anti-CD68 (#EBM11) from Dako (Glostrup, Denmark), anti-MR (#ab8918) from Abcam (Cambridge, United-Kingdom), anti-CD140a (#3174 T) from Cell Signaling (Ozyme, St Quentin en Yvelines, France). Human antibodies for western blot analysis were purchased as indicated: anti-phospho-Smad2 (Ser465/467, #3101) and anti-Smad2/3 (#3102) from Cell Signaling, and anti-β-tubulin from Sigma-Aldrich. Human antibodies against CD56-APC (#341027) and CD140a-PE (#556002) were purchased from BD-Biosciences (Le Pont de Claix, France). Human recombinant IL-4 (#200-04) and IL-1β (#200-01B) were purchased from Peprotech (Neuilly-Sur-Seine, France) and used at 15 ng/ml. SB431542 (#S4317) was purchased from Sigma-Aldrich Chimie and used at 5 µM. DRAQ5 fluorescent probe (#62254) was purchased from Thermofisher Scientific and used at 20 µM.

### Human skeletal muscle FAP isolation

Tissue samples were obtained as *res nullius* from surgeries or diagnostic biopsies from healthy donors aged from 1 to 8 years and with the informed consent of the parents. All protocols for healthy skeletal muscles were approved by the Centre Hospitalier Universitaire de Nice Review Board, according to the French Regulatory Health Authorities. Samples were placed in F10 medium and transferred to the laboratory. DMD biopsies were obtained from Myobank-AFM Institut de Myologie, Paris, France. Characteristics of healthy and DMD muscles are reported in Table 1. Skeletal muscle cells were isolated by a standard method^28^. Briefly, healthy skeletal muscles were minced into 1 mm^3^ fragments and digested at 37 °C, first using liberase (Roche Diagnostics, Meylan, France) for 1 hour and then using 0.25% trypsin-EDTA (Lonza, Verviers, Belgium) for 20 minutes. The enzymatic reaction was stopped by adding 10% fetal bovine serum (FBS). The suspension was homogenized, cells pelleted by centrifugation and cultured in growth culture medium (Ham’s F10 medium supplemented with 20% FBS, 10 mM Hepes, 10^−6^ M dexamethasone, 2.5 ng/ml basic fibroblast growth factor, 100 U/ml penicillin, and 100 mg/ml streptomycin).

**Table 1:** Characteristics of healthy and DMD muscular biopsies.

Name	Gender	Age (year)	Muscle origin
**Healthy muscles**			
1	male	8	paravertebral
2	male	1	paravertebral
3	male	4	paravertebral
4	female	17	paravertebral
5	male	19	paravertebral
6	male	17	gluteus maximus
7	female	15	paravertebral
8	female	16	paravertebral
**DMD muscles**			
1	male	14	latissimus dorsi
2	male	16	gluteus maximus
3	male	15	deltoid
4	male	14	paravertebral
5	male	13	paravertebral
6	male	11	Tensor fasciae latae
7	male	15	paravertebral
8	male	16	paravertebral

FAP purification was performed as previously described^28^. Adherent cells were sorted by flow cytometry with the BD FACSARIA II sorter equipped with 4 lasers and FACSDiva software (Becton, Dickinson and Company, Franklin Lakes, NJ, USA). For CD140a-PE, fluorescence was excited with the 561 nm laser and measured with a 586/15 bandpass filter. For CD56-APC, fluorescence was excited with the 633 nm laser and measured with a 670/14 bandpass filter. Unlike myogenic progenitors, FAPs are negative for myogenic marker CD56 and positive for CD140a expression.

### Human peripheral blood mononuclear cell isolation and culture

Blood mononuclear cells were isolated from healthy donors (French Blood Service, Marseille, France). After Ficoll gradient centrifugation, the monocytes were suspended in RPMI 1640 medium (Gibco, Thermofisher Scientific) containing 0.1 mg/mL gentamycin, 2 mM glutamine and 10% decomplemented human serum. Cells were cultured at a density of 10 × 10^6^ cells/well in 100 mm plastic culture dishes (Corning® Primaria™). Resting macrophages (RM) were obtained from adherent monocytes cultured for 6 days. To induce pro-inflammatory (M(IL-1β)) or anti-inflammatory (M(IL-4)) macrophage phenotype, recombinant human IL-1β or IL-4 was added at the beginning of differentiation, respectively. Conditioned media (CM) from RM, M(IL-1β) or M(IL-4) macrophages were obtained by washing differentiated cells with PBS 1X and then by adding serum and cytokine-free RPMI 1640 medium for an additional 24 hour. At the end, RM, M(IL-1β) and M(IL-4) CM were collected, centrifuged at 1500 rpm for 15 minutes and stored at −80 °C until use.

### FAP and macrophage indirect co-cultures

FAP cells were seeded in 12 well-plastic culture dishes at a density of 1 × 10^5^ cells/well in the growth medium. Two days later, adipogenic differentiation was induced on confluent FAPs by switching the growth medium to a pro-adipogenic differentiation medium (Ham’s F10/F12/low-glucose DMEM with 2 mM glutamine (2 v/1 v/1 v), 2% horse serum, 1 mM dexamethasone, 100 µM 1-methyl-3-isobutylmethyl-xanthine (MIX), 15 µg/ml insulin, 15 µg/ml transferrin, 0.2 nM triiodothyronine, and 100 nM rosiglitazone) containing RM, M(IL-1β) or M(IL-4) CM (at the ratio of 50/50). Three days later, cells were placed in the same medium lacking MIX and dexamethasone but still containing CM at the ratio 50/50. This differentiation medium was replaced every 3 days and cells were collected after 10 days of differentiation.

### Oil red O staining and adipocyte morphometry analyses

Differentiated adipocytes were PBS-washed, fixed in 4% paraformaldehyde for 10 min, and treated for 30 min with oil red O (60% of a stock solution at 0.5% w/v in isopropanol and 40% distilled water). Cells were washed with isopropanol/water (6 volumes/1 volume) to remove the unspecific staining and then several times with water. Images were recorded with a TE-2000U bright-field optical microscope (Nikon, Tokyo, Japan). Lipid droplet areas were analyzed using ImageJ software (National Institutes of Health, Bethesda, MD, USA). The number of adipocytes *per* field was counted on at least 15 different fields. The number of lipid droplets was counted *per* adipocyte. Incomplete droplets located at the edge of the image were excluded. At least 30 adipocytes from each condition were measured.

### Immunofluorescence and histological staining

Cryosections of human healthy and DMD muscles or cultured cells were fixed with Histofix 4% (Carl Roth, Lauterbourg, France) for 10 minutes, permeabilized and saturated with 0.1% Triton X-100/3% bovine serum albumin for 30 minutes, subsequently incubated over night at 4 °C with primary antibody, and then with secondary antibody Alexa Fluor 594 or 488 goat anti-mouse or anti-rabbit IgG (Molecular Probes) for 45 minutes at room temperature. Samples were finally mounted in Mowiol containing DAPI and visualized with an Axiovert miscroscope (Carl Zeiss, Le Pecq, France) under oil immersion. Images were captured and analysed with AxioVision software (Carl Zeiss; Le Pecq, France).

### Second Harmonic Generation (SHG) imaging

Imaging was performed on an LSM 780 NLO inverted Axio Observer.Z1 confocal microscope (Carl Zeiss Microscopy GmbH, Jena, Germany) using a Plan Apo 25X multi-immersion (oil, glycerol, water) NA 0.8 lense. Fluorescence images were acquired using a laser 561 nm for Alexa 594 and a laser 640 nm for DRAQ5. The SHG light source was a Mai Tai DeepSee (Newport Corp., Irvine, CA, USA) tuned at 880 nm. Forward SHG signals were detected with an Oil condenser (1.4 NA), 440/40 nm bandpass filter and transmission PMT. Backward SHG was collected on GaAsP (BIG) non-descanned module with 440/10 nm (internal control, not shown). 1 pxl = 168 nm.

### RNA extraction and Reverse Transcription quantitative polymerase chain reaction (Q-PCR)

Total RNA was extracted using TRI-reagent (Euromedex, Souffelweyersheim, France). Methods of RNA extraction and quantitative Q-PCR were previously described^28^. TATA box-binding protein (*TBP*) was used as housekeeping gene. The sequences of primers used are listed in Table 2.

**Table 2:** Q-PCR primers.

Name	Forward primer	Reverse primer
Quantitative PCR primers		
*ACTA2*	TGCCTGCATGGGCAAGTGA	CTGGGCAGCGGAAACG
*ADIPOQ*	GCAGTCTGTGGTTCTGATTCCATAC	GCCCTTGAGTCGTGGTTTCC
*CCL2*	CCCCAGTCACCTGCTGTTAT	TGGAATCCTGAACCCACTTC
*CCL18*	AGCTCTGCTGCCTCGTCTAT	CCCACTTCTTATTGGGGTCA
*CD200R1*	CATCGTGGATATCACCTCCAA	CTTGCTTAGTGGCACAATCGC
*C/EBPA*	CTTGTGCCTTGGAAATGCAAATGCAA	GCTGTAGCCTCGGGAAGGA
*C/EBPB*	AACCAACCGCACATGCAGAT	GGCAGAGGGAGAAGCAGAGAGT
*COL1A1*	ACCTGCGTGTACCCCACTCA	CCGCCATACTCGAACTGGAA
*CXCL8*	AGGAAGAAACCACCGGAAGG	GGCAAAACTGCACCTTCACA
*F13A1*	CTGACCTTCCTGTTGGATTTG	CTTGATGGCTTGAACCGAGG
*FABP4*	ATGGGATGGAAAATCAACCA	TGCTTGCTAAATCAGGGAAAA
*FN1*	CTGGCCGAAAATACATTGTAAA	CCACAGTCGGGTCAGGAG
*IL1B*	AGCTCGCCAGTGAAATGATGG	CAGGTCCTGGAAGGAGCACTTC
*IL6*	GGCACTGGCAGAAAACAACC	GCAAGTCTCCTCATTGAATCC
*LEP*	AGGGAGACCGAGCGCTTTC	TGCATCTCCACACACCAAACC
*MRC1*	GCAATCCCGGTTCTCATCGC	CGAGGAAGAGGTTCGGTTCACC
*PLIN1*	ACCATCTCCACCCGCCTC	GATGGGAACGCTGATGCTGT
*PPARG*	AGCCTCATGAAGAGCCTTCCA	TCCGGAAGAAACCCTTGCA
*TBP*	ACGCCAGCTTCGGAGAGTTC	CAAACCGCTTGGGATTATATTCG
*TNFA*	GCCCATGTTGTAGCAAACCC	TATCTCTCAGCTCCACGCCA

### Western blot analysis

Cells were lysed in RIPA buffer consisting of 50 mM Tris-HCl pH 8.0, 150 mM NaCl, 0.1% SDS, 0.5% sodium deoxycholate, 5 mM NaF, 2.5 mM Na_4_P_2_O_7_, 1% NP40, 2 mM sodium vanadate and protease inhibitor cocktail (Roche Diagnostics, Meylan, France). Cell lysates were centrifuged at 13 000 g for 10 minutes at 4 °C, the supernatants were recovered and the protein content determined (Pierce BCA Protein Assay Kit, Thermoscientific, Rockford, IL, USA; #23227). 10 µg of proteins were resolved by 7.5% SDS-PAGE under reducing conditions and transferred to Immobilon-P membranes (Millipore Corporation, Bedford, MA, USA). The membranes were probed with primary antibodies that were detected by horseradish peroxidase-conjugated secondary antibodies (Promega, Charbonnières-les-bains, France) and visualized with an electrochemical luminescence detection kit (Bio-Rad, Marnes-la-Coquette, France). Band intensities were measured by the Quantity One software (Bio-Rad).

### Statistical analysis

Statistical differences between groups were evaluated using the two-tailed unpaired Student’s t-test. A P-value < 0.05 was considered as significant.

## Electronic supplementary material

Supplementary figures
