# Supplementary material for: IL-1β- and IL-4-polarized macrophages have opposite effects on adipogenesis of intramuscular fibro-adipogenic progenitors in humans
Source: Sci Rep. 2018 Nov 19;8:17005. doi: 10.1038/s41598-018-35429-w (PMC6242986; doi:10.1038/s41598-018-35429-w)
Supplement: Supplementary file 1 — Supplementary figures [file 41598_2018_35429_MOESM1_ESM.pdf]

Supplementary Figures

IL-1β- and IL-4-polarized macrophages have opposite effects on adipogenesis of intramuscular fibro-adipogenic progenitors in humans

C Moratal, J Raffort, N Arrighi, S Rekima, S Schaub, CA Dechesne, G Chinetti and C Dani

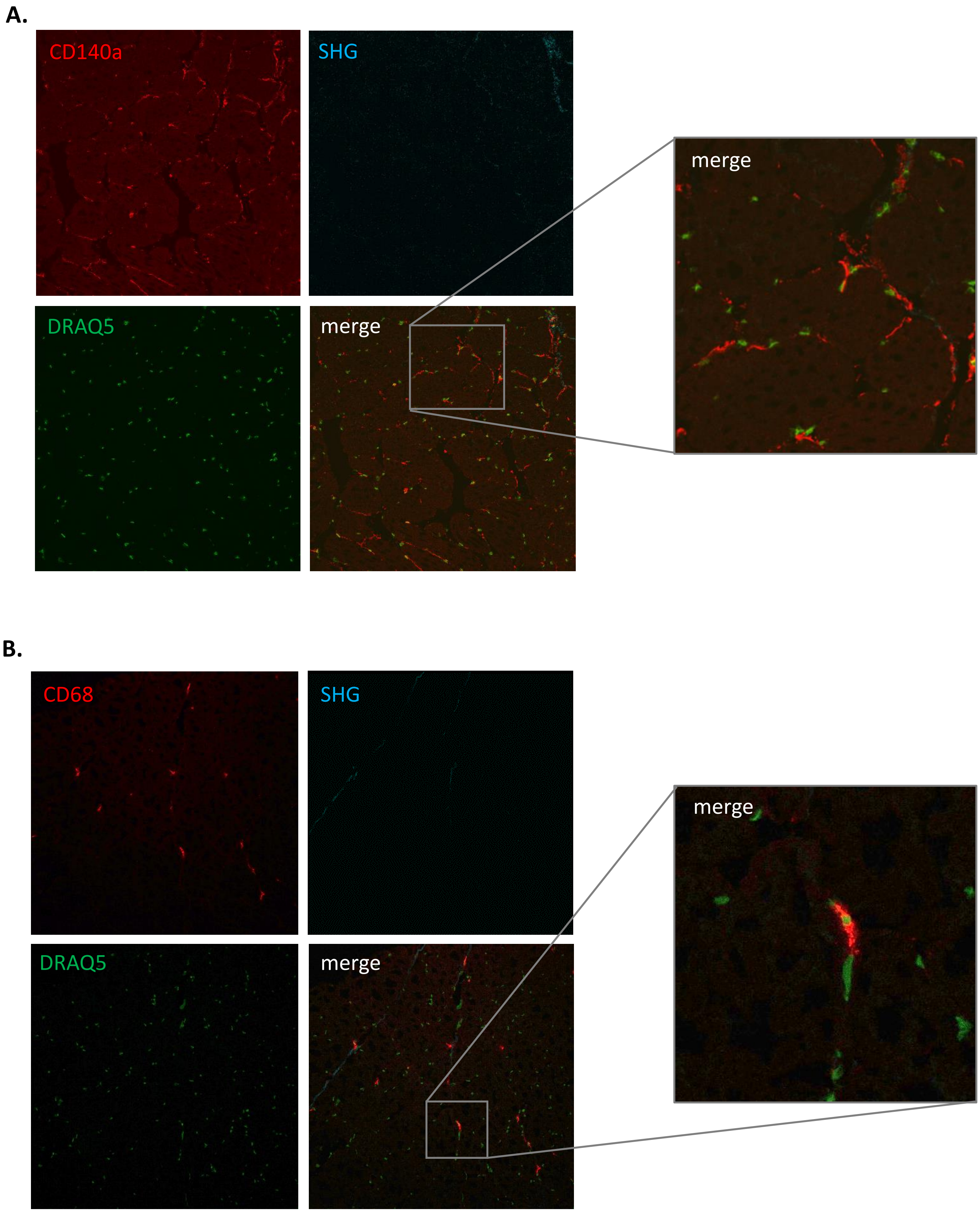

Supplementary figure 1. CD140a and CD68 immunostaining in healthy muscle biopsies. Frozen sections of healthy biopsies were stained in red with anti-CD140a for FAPs *(A)*, or with anti-CD68 for macrophages *(B)*. Fibrillar collagen was visualized in blue by second-harmonic generation imaging (SHG). DNA was marked in green with the fluorescent probe DRAQ5. Representative views are shown. The analysis was performed on two healthy biopsies.

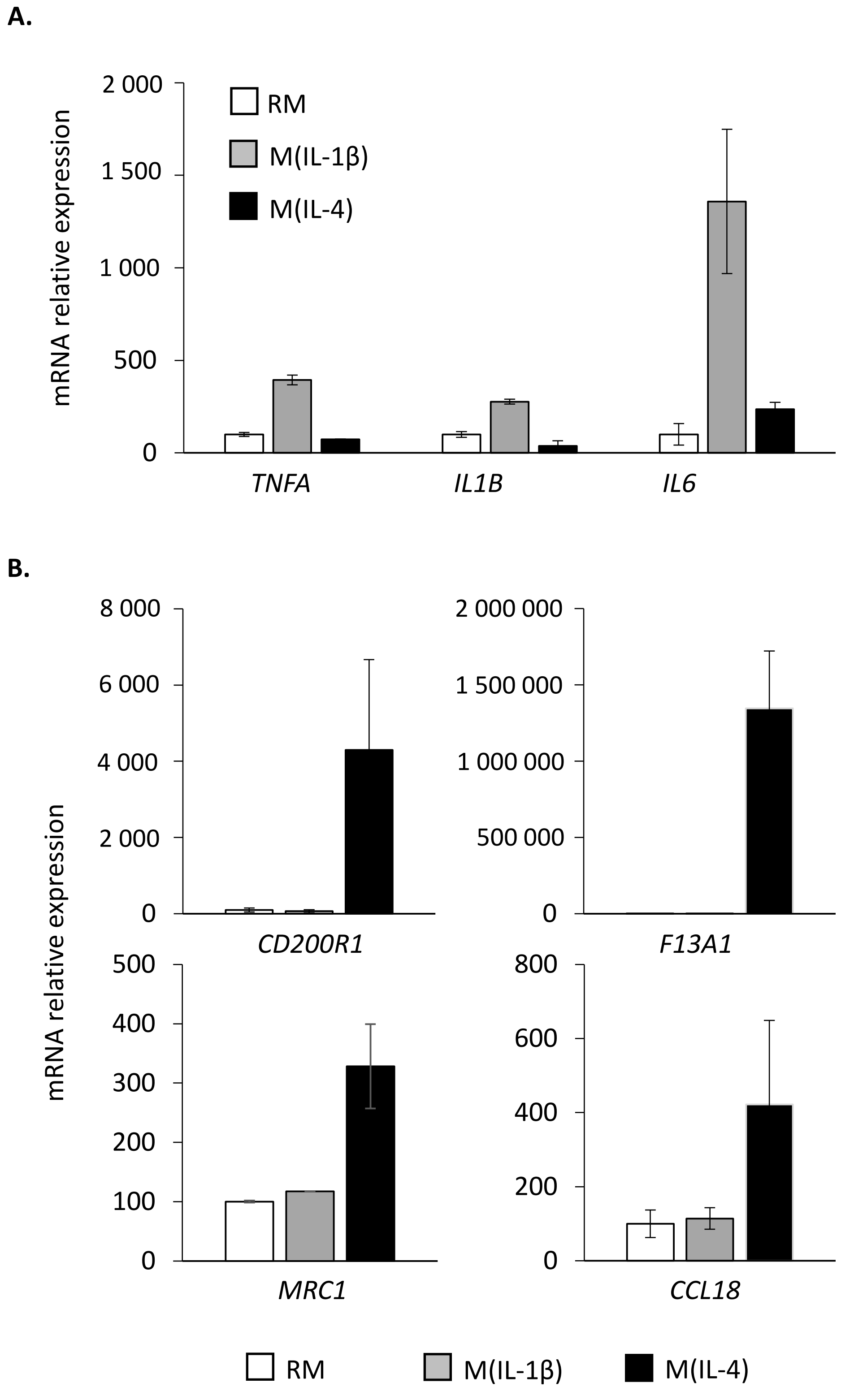

Supplementary figure 2. Characterization of IL-1β- and IL-4-polarized human primary macrophages. Circulating monocytes isolated from healthy donors were polarized in the presence of medium containing 10% of human serum supplemented or not (RM) with 15 ng/ml of IL-1β (M(IL-1β)) or 15 ng/ml of IL-4 (M(IL-4)) for 6 days. Expression of pro-inflammatory (*A*) or anti-inflammatory (*B*) markers was measured by quantitative Q-PCR. Data are presented as means ± standard deviation of duplicates from one blood donor. The experiment was replicated on two other blood donors.

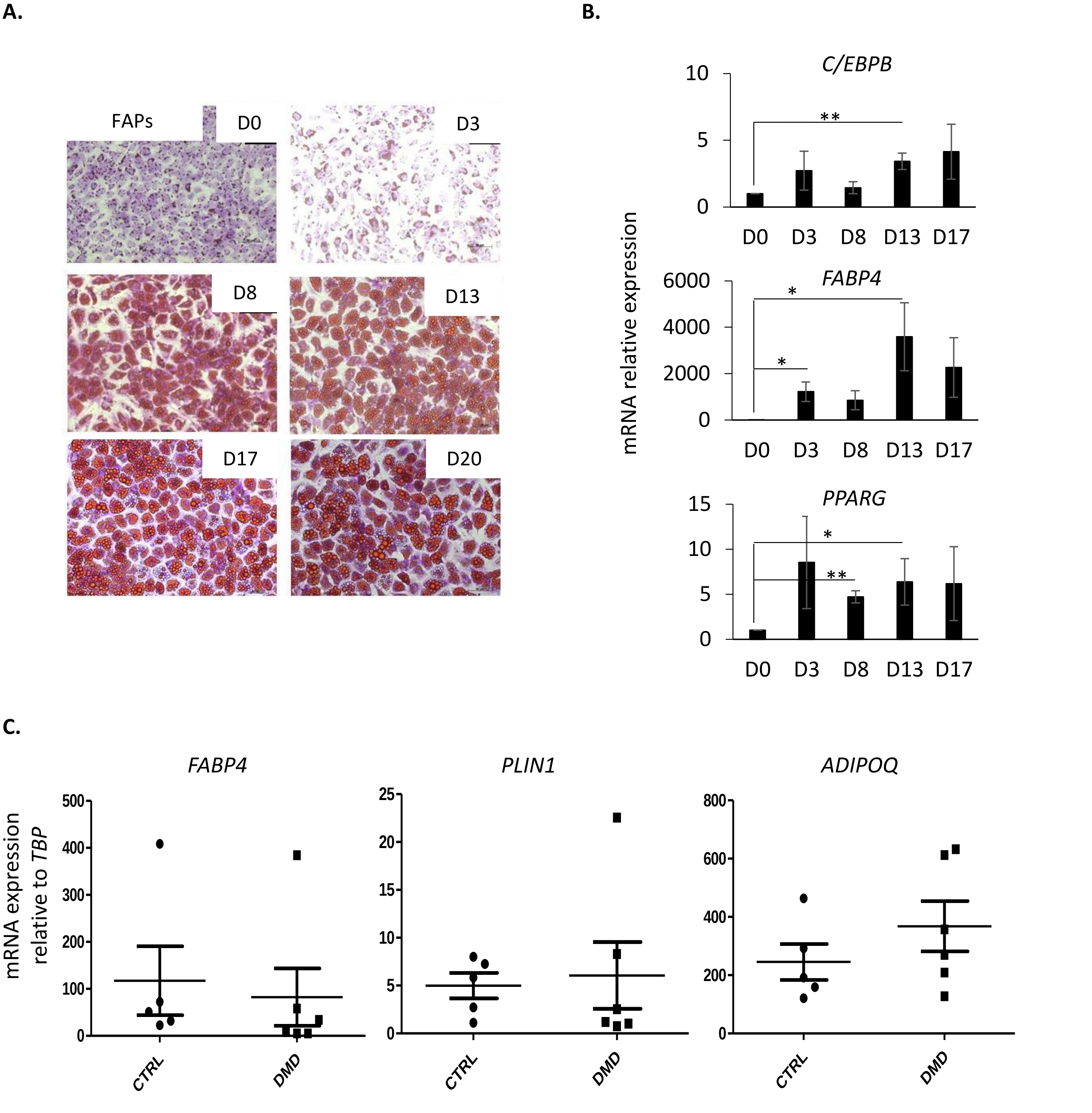

Supplementary figure 3. Adipogenic differentiation rate of FAPs. Freshly FAPs isolated from four healthy donors were plated and two days later, when FAPs were confluent, their differentiation into adipocytes was induced by a pro-adipogenic medium. Analysis of adipogenesis was evaluated before the induction of differentiation (D0) or after 3 (D3), 8 (D8), 13 (D13), 17 (D17) or 20 days (D20) of differentiation. *A*) Adipocytes were stained by oil Red O. Pictures were captured under light microscopy. *B) FABP4*, *PPARG* and *C/EBPB* gene expression was measured by quantitative Q-PCR. Data are presented as means ± SEM of three separate experiments in duplicates; * $P < 0.05$; ** $P < 0.01$ *vs* D0. *C)* Freshly FAPs isolated from five healthy donors (CTRL) or from five DMD patients (DMD) were differentiated into adipocytes in a pro-adipogenic medium for 10 days. *PLIN1*, *FABP4* and *ADIPOQ* gene expression was measured by quantitative Q-PCR.

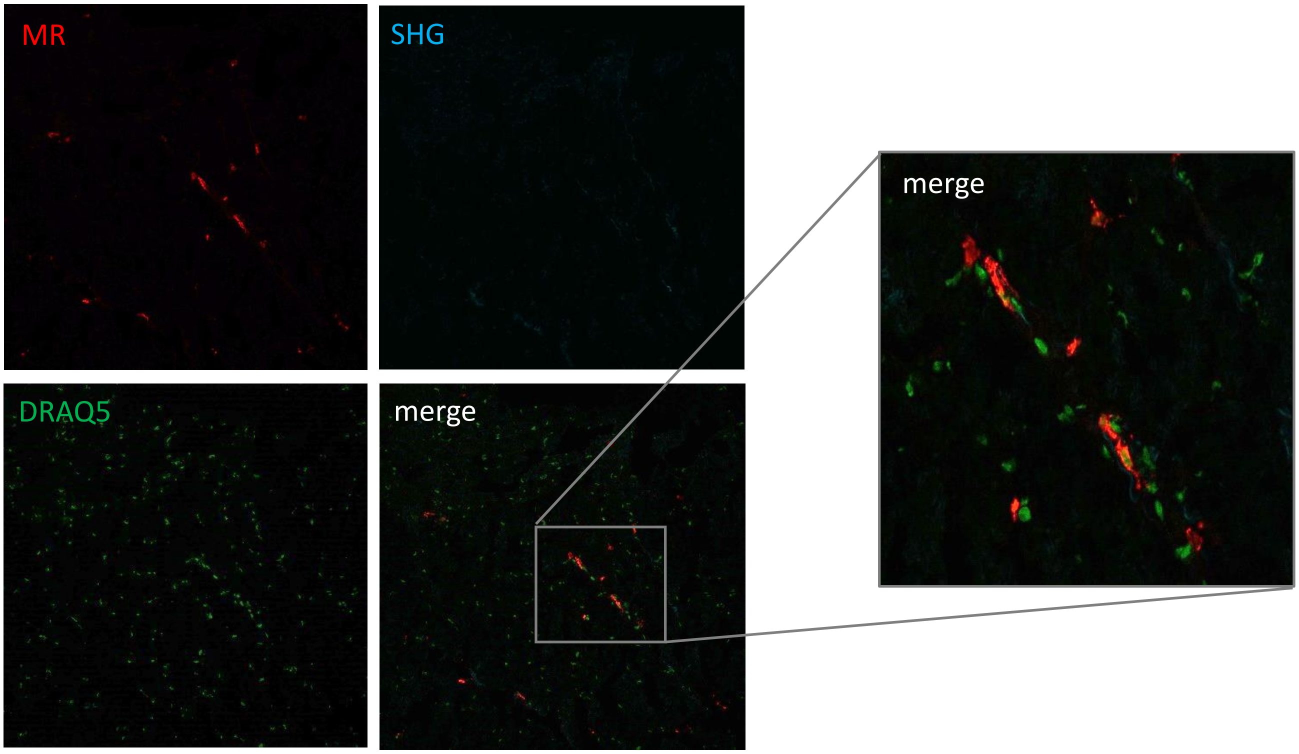

Supplementary figure 4. MR immunostaining in healthy muscle biopsies. Frozen sections of healthy biopsies were stained in red with anti-MR for macrophages. Fibrillar collagen was visualized in blue by second-harmonic generation imaging (SHG). DNA was marked in green with the fluorescent probe DRAQ5. Representative views are shown. The analysis was performed on two healthy biopsies.

Original pictures from Fig. 4E

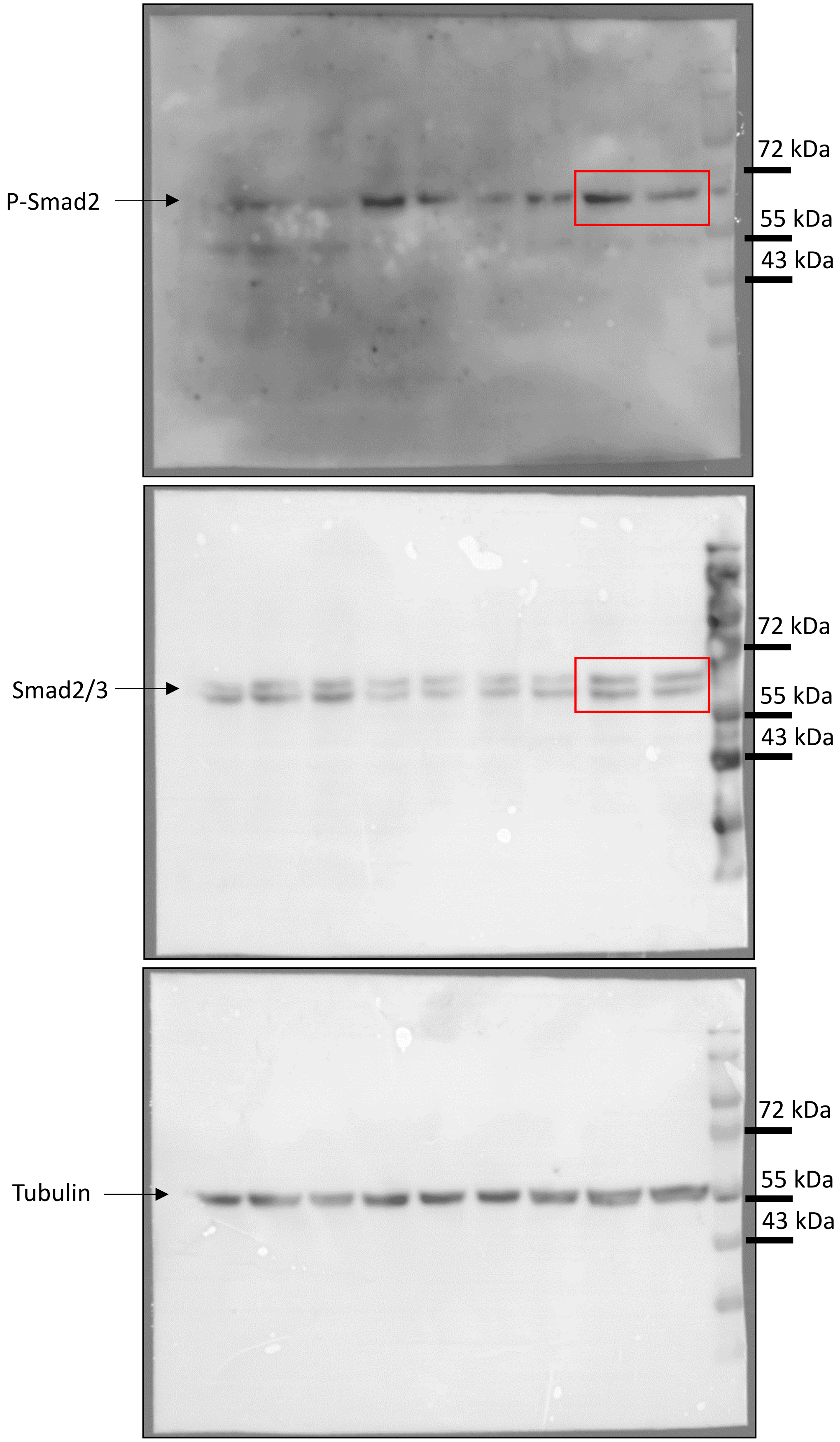

This figure represents the whole images of the Western Blot taken by a Biorad Chemidoc XRS+ imaging system. The molecular weights and the antibodies used are indicated on the Western-Blots. The red frames show the views used in Fig. 4E.
